# Identification of a novel cord blood NK cell subpopulation expressing functional programmed death receptor-1

**DOI:** 10.3389/fimmu.2023.1183215

**Published:** 2023-06-22

**Authors:** Marco Greppi, Valentina Obino, Rayan Goda, Federico Rebaudi, Simona Carlomagno, Mariella Della Chiesa, Simona Sivori, Gianluca Ubezio, Vanessa Agostini, Alessandra Bo, Silvia Pesce, Emanuela Marcenaro

**Affiliations:** ^1^ Department of Experimental Medicine, University of Genoa, Genoa, Italy; ^2^ Department of Medicine (DAME), University of Udine, Udine, Italy; ^3^ IRCCS Ospedale Policlinico San Martino, Genova, Italy

**Keywords:** programmed death receptor 1 (PD-1), human natural killer (NK) cells, cord blood (CB), killer ig-like receptor (KIR), NKG2A, NK cell maturation, immune checkpoint, human cytomegalovirus (CMV)

## Abstract

**Background:**

Natural Killer cells (NKs) represent the innate counterpart of TCRαβ lymphocytes and are characterized by a high anti-tumor and an anti-viral cytotoxic activity. Recently, it has been demonstrated that NKs can express PD-1 as an additional inhibitory receptor. Specifically, PD-1 was identified on a subpopulation of terminally differentiated NKs from healthy adults with previous HCMV infection. So far it is unknown whether PD-1 appears during NK-cell development and whether this process is directly or indirectly related to HCMV infection.

**Methods:**

In this study, we analyzed the expression and function of PD-1 on Cord Blood derived NKs (CB-NKs) on a large cohort of newborns through multiparametric cytofluorimetric analysis.

**Results:**

We identified PD-1 on CB-NKs in more than of half the newborns analyzed. PD-1 was present on CD56^dim^ NKs, and particularly abundant on CD56^neg^ NKs, but only rarely present on CD56^bright^ NKs. Importantly, unlike in adult healthy donors, in CB-NKs PD-1 is co-expressed not only with KIR, but also with NKG2A. PD-1 expression was independent of HCMV mother seropositivity and occurs in the absence of HCMV infection/reactivation during pregnancy. Notably, PD-1 expressed on CB-NKs was functional and mediated negative signals when triggered.

**Conclusion:**

To our understanding, this study is the first to report PD-1 expression on CB derived NKs and its features in perinatal conditions. These data may prove important in selecting the most suitable CB derived NK cell population for the development of different immunotherapeutic treatments.

## 1 Introduction

Recently, several mechanisms that may compromise NK-mediated anti-tumor activity have been described. These include the expression of different inhibitory checkpoints (ICs), such as the HLA-specific receptors KIRs, LILRB-1 and NKG2A, in addition to other non-classical inhibitory receptors, such as PD-1 (whose ligands are the molecules PD-L1 and PD-L2) (1, 2).

These checkpoints, primarily play an important role in the immune response regulation and in maintaining tolerance also being implicated in the education/licensing processes of NK cells during their development (3). However, it is now well known that they can be co-opted by tumor cells expressing the specific ligands, and their engagement can lead to inhibition of the anti-tumor activity of NKs, allowing tumor proliferation in an uncontrolled manner (4). In this context, novel immunotherapies based on monoclonal antibody (mAb)-mediated blockade of these ICs have been developed to restore or increase the anti-tumor cytotoxic activity of these cells (4–8).

In humans, NKs are not a homogeneous population, but based on the surface density of CD56, they are divided into two main subpopulations:

-CD56^bright^, which represent 10% of the peripheral blood (PB) NKs, are defined as more immature cells and have a greater proliferative capacity, but are less cytotoxic;
-CD56^dim^, which represent the remaining 90%, are considered the next maturation step and have greater cytolytic activity.

Numerous studies indicate that CD56^bright^ and CD56^dim^ subpopulations represent two sequential differentiative stages of NKs. It is thought that CD56^bright^ NKs can differentiate into CD56^dim^ NKs (9, 10). CD56^dim^ cells can continue to differentiate during their lifetime by acquiring new phenotypic and functional properties. These cells comprise a fraction of terminally differentiated cells that are KIR^+^NKG2A^-^ and are characterized by the expression of CD57. This molecule is virtually absent at the fetal period and at birth, whereas it increases with age. All cellular intermediates of this maturation process are represented in varying proportions in the “steady state” and appear, over time, during immune reconstitution, as demonstrated in humanized mice and in patients undergoing hematopoietic stem cell transplantation (HSCT) (11).

In addition to the emergence of high percentages of CD56^neg^CD16^+^ NKs, HCMV infection induces a strong imprinting in the development of NKs not only by accelerating the differentiation of NKG2A^-^KIR^+^ NKs, but also by inducing a significant increase in CD56^dim^ NKs expressing the NKG2C receptor together with CD57: the so-called adaptive NKs (11, 12)

The CD56^neg^ population is abundant in the cord blood (CB), even though its presence cannot be correlated to viral infections. Some works indicated that this population may represent a subset of more immature cells (13), however these claims are not supported by the phenotype of these cells expressing high levels of KIRs. So, it is still to be clarified why this population is so represented in newborns.

Recently, it has been shown that some individuals can express *de novo* another receptor we mentioned earlier: PD-1. Specifically, this receptor has been identified in adult healthy donors (AHD) seropositive for HCMV (18-60 years), and its expression is confined to terminally differentiated NKs (CD56^dim^, KIR^+^, NKG2A^-^, CD57^+^) and, if present, on CD56^neg^ NKs, whereas it is absent on more immature NKs (CD56^bright^ and CD56^dim^NKG2A^+^) (1).

So far, it is unknown if and how PD-1 is involved in NK cell development, maturation, and whether this process is directly or indirectly related/connected to HCMV infection, or if its expression can be linked to the NK cell “education” process.

To answer these questions, we analyzed the phenotypic and functional features of NKs in newborns’ CB. Our results demonstrate that, at birth, we can find newborns with PD-1 on NKs, co-expressing NKG2A and KIRs and this expression is completely independent of HCMV infection.

PD-1 expression enhances CB-NK cell effector functions while inhibits them upon interaction with PD-Ls^+^ target cells, suggesting a possible role for this receptor in the NK cells’ education process.

## 2 Materials and methods

### 2.1 Samples

This study was conducted using NKs derived from mothers’ peripheral blood and the respective CB obtained from voluntary umbilical cord donations, received at the cord donation center of IRCCS Ospedale Policlinico S. Martino, Genova. NKs derived from voluntary blood donors received at the transfusion center of IRCCS Ospedale Policlinico S. Martino, Genova, Italy, were used as control. All biological samples were collected after obtaining informed consent from the donors in accordance with the Declaration of Helsinki. The study was approved by the Ethics Committee of the Region Liguria (Prot. n. 39/2012, number CER Liguria: DB id 10125).

### 2.2 Cell preparation and cytofluorimetric analysis

Mononuclear cells derived from heparinized CB and PB were obtained by centrifugation on Ficoll gradient (Sigma, St. Louis, MO) and then resuspended in RPMI 1640 medium supplemented with 2mM glutamine, 50 ug/mL penicillin, 50 ug/mL streptomycin and 10% heat-inactivated FCS (Fetal Calf Serum, Biochrom, Berlin, Germany).

Cytofluorimetric analyses were performed using the FACS Verse cytofluorimeter (Becton Dickinson, Mountain View, CA). The data were analyzed with the FACSuite (Becton Dickinson, Mountain View, CA) and Flowjo software. We also used FlowJo v10 for visualization of the unbiased t-distributed stochastic neighbor-embedding (t-SNE) algorithm. The constructed sample was generated concatenating 1000 PD-1^+^ NKs and 1000 PD-1^-^ NKs derived from 15 different CB samples using the concatenate function of FlowJo™ v10.8 Software (BD Life Sciences), this sample was represented as a t-SNE generated using the automatic learning configuration with parameters defined by Belkina et al. (14) and a perplexity value of 30.

Results on PD-L1 and PD-L2 expression on monocytes and NKs were interpreted with a unsupervised hierarchical clustering analysis using the online resource Morpheus (15) with the following settings: Metric: Euclidean distance; Linkage Method: Average

### 2.3 Monoclonal antibodies

The following antibodies were isolated in our laboratory, approved by the indicated companies, and validated for their specificities: anti-NKp30 (AZ20, IgG1), anti-NKp46 (BAB281, IgG1); Beckman Coulter/Immunotech, Marseille, France, anti-Siglec-7 (QA79, IgG1); R&D Systems, Abingdon, United Kingdom. For the following antibodies the specificities have been validated in the corresponding patent or assigned in a group in CD workshop: anti-LILRB-1 (F278, IgG1), KIR3DL1/L2-S1 (AZ158, IgG2a). The purified anti-PD-1 mAbs (PD-1.3.1.3 clone, IgG2b), the purified anti–PD-L1 (PDL1.3.1 clone, IgG1) and the purified anti–PD-L2 (326.35 clone, IgG1 were originally isolated from Laboratoire Immunologie des Tumeurs, CRCM, Marseille-Luminy (France). Other antibodies used in this study were: anti-CD56-PC7 (clone N901), and anti-NKG2A-APC (clone Z199) bought from BeckmanCoulter/Immunotech; anti-CD16-PerCP-Cy5. 5 (clone 3G8), anti-KIR2DL2/L3-S2-FITC (CH-L clone) and anti-CD107a–PE (anti-LAMP1), from BD Biosciences PharMingen; and anti-KIR2DL1-S1 FITC (11PB6 clone), anti-KIR3DL1 FITC (DX9 clone), anti-NKG2C VioBright FITC, anti-CD3-VioGreen (BW264/56 clone), anti-CD19-VioGreen (LT19 clone), and anti-CD57-VioBlue (TB03 clone) mAbs were purchased from Miltenyi Biotec.

Isotype-specific goat anti-mouse secondary antibodies were purchased from Southern Biotechnology (Birmingham, Ala) and, in the case of APC IgG2b, by Jackson ImmunoResearch (Newmarket, United Kingdom).

For R-ADCC and masking experiments, additional mAbs isolated in our laboratory and validated for their specificity were used: anti–HLA class I (A6/136, IgM) and anti-CD16 (c127, IgG1).

### 2.4 Gating strategy

Information on gating strategies is shown in **Figure S1** and indicated in each Figure legend.

### 2.5 Statistical analysis

**Figure 1:**
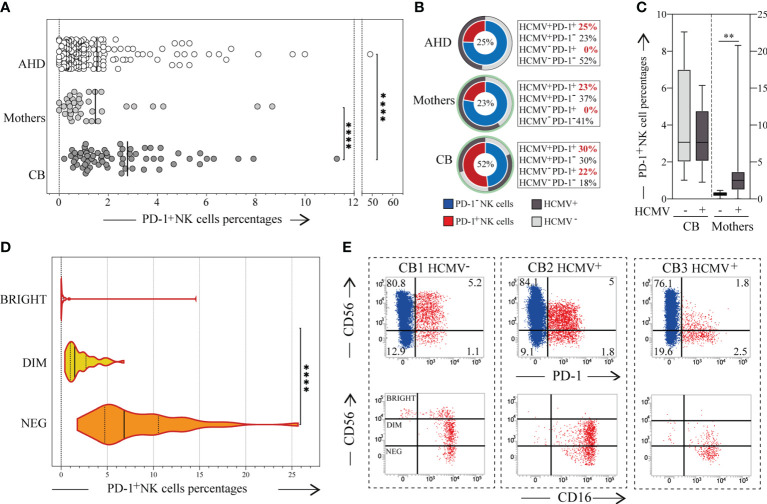
PD-1 expression on CB-NKs. **(A)** Expression of PD-1 on NKs derived from the peripheral blood of Adult Healthy Donors (AHD, white dots; N=200), the peripheral blood of the mothers collected after giving birth (Mother, light gray dots; N=37), and the cord blood of newborns (CB, dark gray dots; N=68); **(B)** Frequency of adult healthy donor (AHD, top), mothers (mother, middle) and Cord Blood (CB, bottom) characterized by a PD-1^+^ NK cell subset (red slice) and/or HCMV seropositivity (dark grey slice), CB’s HCMV seropositivity was defined by mothers HCMV status (light green circle); **(C)** Expression of PD-1 on CB’s and Mothers’ NK cells in relation to mother’s HCMV status; **(D)** Expression of PD-1 on CD56^bright^ (white), CD56^dim^ (yellow) and CD56^neg^ (orange) CB-NKs (N=50); **(E)** Dot plots illustrating PD-1 expression on NKs from three representative CB samples (up), dot plot illustrating CD56 with CD16 expression on PD-1^+^ NK cells (red cells) of the same representative three CB samples (down); Gating Strategy: **(A, D)** I; **(C)** II. P value of less than 0.01 (**), and less than 0.0001 (****) was considered statistically significant; when not indicated, data were not statistically significant.

**Figure 2:**
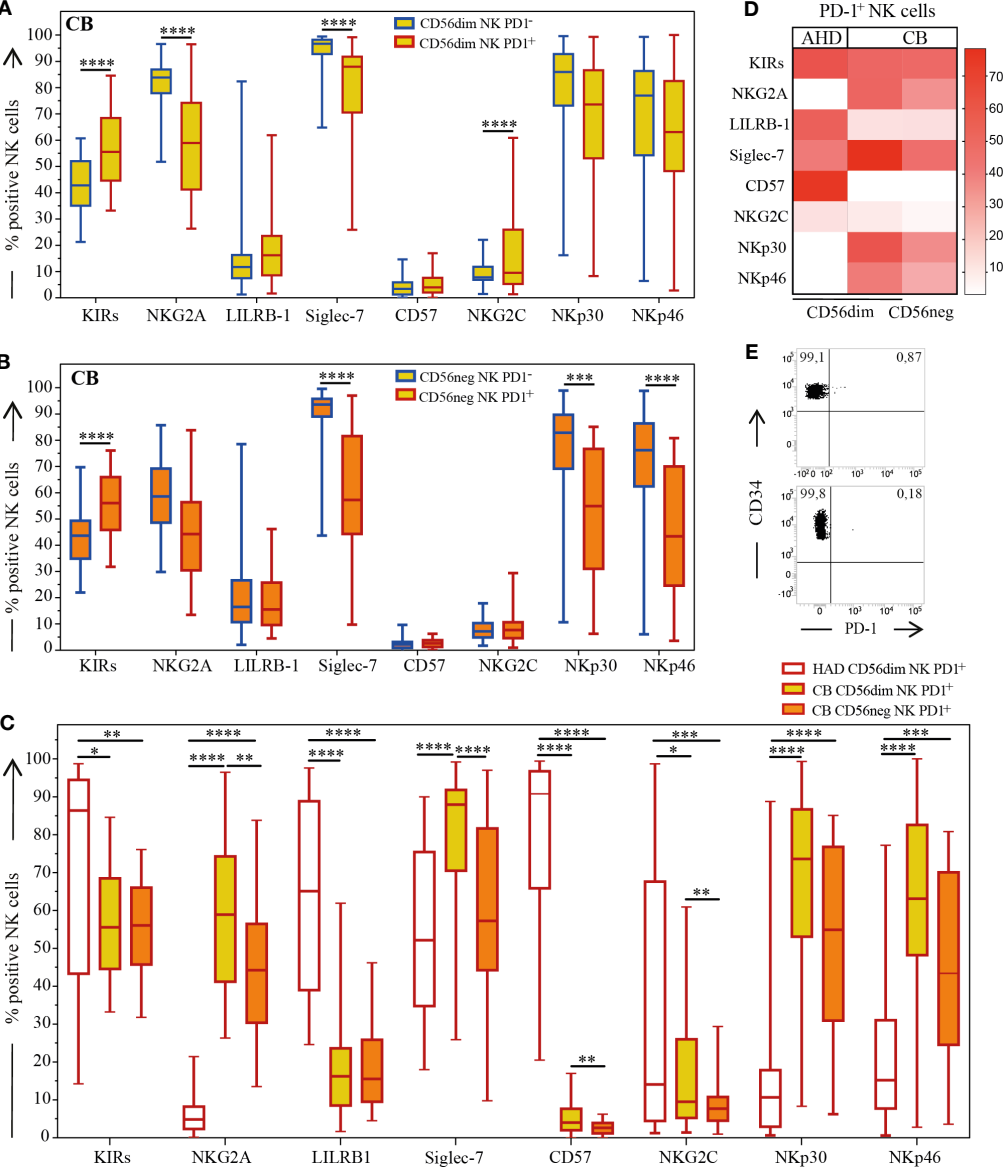
Expression of main NK cell receptors on AHD and CB-NKs. Comparison of main NK cell receptors’ expression on PD-1^+^ (red outline) and PD-1^-^ (blue line) NKs; **(A)** Box and Whisker plot representing CD56dim CB-NKs (top, yellow); **(B)** Box and Whisker plot representing CD56^neg^ CB-NKs (middle, orange); **(C)** Box and Whisker plot and **(D)** Heatmap comparing PD-1^+^ CB-NKs to PD-1 AHD-NKs (bottom); **(E)** Two (out of 7) representative dot plot illustrating PD-1 expression on CD34^+^ cells derived from CB (N=43 for CB and N=200 for AHD). Gating Strategy: Panel A-D: III; Panel E: IV. P value of less than 0.05 (*), less than 0.01 (**), less than 0.001 (***) and less than 0.0001 (****) was considered statistically significant; when not indicated, data were not statistically significant.

**Figure 3:**
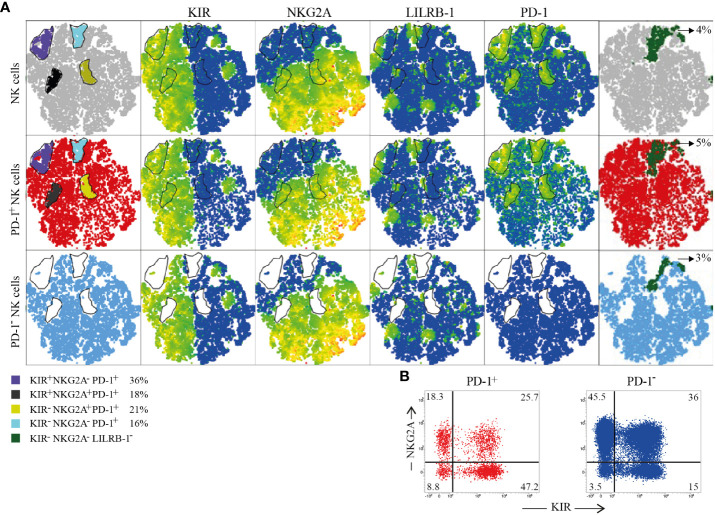
Immune Checkpoints coexpression on PD-1^+^ and PD-1^-^ CB-NKs. **(A)** t-SNE generated from an equal number of PD-1^+^ and PD-1^-^ CB-NKs derived from 15 different CB. The first row shows all CB-NKs, the second row shows only PD-1^+^ CB-NKs, the third row shows only PD-1^-^ CB-NKs. In the first column we highlighted 4 PD-1^high^ NK cell subsets: 1) characterized by the PD-1^high^KIR^+^NKG2A^-^ phenotype (purple), 2) characterized by the PD-1^high^KIRs^-^NKG2A^-^ phenotype (light blue), 3) characterized by the PD-1^high^KIR^+^NKG2A^+^ phenotype (gray), and 4) characterized by the PD-1^high^KIRs^-^NKG2A^+^ phenotype (yellow). The percentages indicated in the legend refer to PD-1^+^ cells. In the last column we highlighted KIRs^-^NKG2A^-^LILRB-1^-^ NKs (dark green). **(B)** Representative dot plot illustrating the distribution of NKG2A and KIR on PD-1^+^ (red) and PD-1^-^ (blu) NK cells. Gating Strategy **(A, B)** V.

Statistical analysis was performed with Graphpad Prism (La Jolla, CA) software. For statistical analysis of cytofluorimetric experiments nonparametric t tests were used: Mann−Whitney for analyses showed in **Figures 1**, **2**, **Figure 3B, C**, and **Figure S2**; Wilcoxon matched-pairs signed rank test for analyses showed in **Figures 3D–F**. P value of less than 0.05 (*), less than 0.01 (**), less than 0.001 (***) and less than 0.0001 (****) was considered statistically significant; when not indicated, data were not statistically significant.

### 2.6 ELISA

HCMV seropositivity was detected by using Vironostika anti-CMV III (bioMerieux, Grenoble, France), an ELISA for the detection of total antibody to CMV in human serum. Soluble PD-L1 was evaluated with Human PD-L1 DuoSet ELISA (R&D System; Catalog #: DY156) following the kit procedure.

### 2.7 Degranulation assay and IFN-γ production

These experiments were performed by evaluating gated PD-1^+^ or PD-1^-^ CB-NK cell subsets for degranulation (based on surface expression of CD107a), and IFN-γ production, upon interaction with different target cells. For degranulation assay, CB cells were cultured overnight (O.N.) in the presence of low doses of rhIL-15 (0.5 ng/mL) and then co-incubated with target cells at an E/T ratio of 2:1 in a final volume of 200 μl in round-bottomed 96-well plates at 37°C and 5% CO2 for 3h in culture medium supplemented with anti-CD107a-PE mAb with different target cells. We used OVCAR-5 human ovarian carcinoma cell line for masking experiments and FcγR^+^P815 murine mastocytoma cell line for reverse antibody-dependent cell cytotoxicity (R-ADCC). The cocultures with OVCAR5 cell line were performed in the presence of anti–HLA class I (A6/136, IgM) mAb in combination or not with anti–PD-L1 (PDL1.3.1 clone, IgG1) and anti–PD-L2 (326.35 clone, IgG1) mAbs; the cocultures with FcγR^+^P815 cell line were performed in the presence of anti-CD16 mAb (c127, IgG1) in combination or not with anti-PD-1 mAb (PD-1.3.1.3 clone, IgG2b) (R-ADCC), as indicated in the appropriate Figures.

Cells were then washed, stained first with anti-PD-1 mAb for 30 minutes at 4°C, then washed and stained with IgG2b-PE secondary antibody for 30 minutes at 4°C and finally after two washes, cells were stained with anti-CD3, anti-CD56, anti-CD19, anti-CD14, anti-NKG2A, anti-KIRs (11PB6, CH-L, DX9 clones) mAbs for 30 minutes at 4°C, washed, acquired by flow cytometry (BD FACSVerse and analyzed by FlowJo™ v10.8 Software (BD Life Sciences).

For detection of IFN-γ production, CB cells were cultured O.N. in the presence of low doses of rhIL-15 (0.5 ng/mL), washed, and labeled for 5 minutes at a concentration of 10x10^6^ cells/ml in ice-cold medium with 50 μg/ml anti-IFN-γ/CD45 Ab-Ab conjugates (Miltenyi Biotec). Cells were suspended in 37°C medium to a final concentration of 10x10^6^ cells/ml and were allowed to secrete IFN-γ in culture with FcγR^+^P815 murine mastocytoma cell (E/T ratio: 2:1), in the presence of anti-CD16 mAb (c127, IgG1) in combination or not with anti-PD-1 mAb (PD-1.3.1.3 clone, IgG2b), as indicated in the appropriate Figure, for 3 h at 37°C and 5% CO2 under slow

continuous rotation using the MACSmix Tube Rotator (Miltenyi Biotec). After capturing secreted cytokines at their surface, cells were centrifuged at 300 g for 5 minutes at 4°C and resuspended at a concentration of 10x10^6^ cells/ml in ice-cold PBS containing 0.5% BSA and 5 mM EDTA (both from Sigma-Aldrich). The cells were then stained with 5 μg/ml PE-conjugated anti-IFN-γ for 10 min at 4°C.

Cells were then washed, stained, and analyzed following the same protocol used for degranulation assay samples (16).

## 3 Results

### 3.1 Cord blood NKs express PD-1

We analyzed the expression of PD-1 on NKs in 68 newborns’ CB (CB-NKs) and in their relative mothers. We detected the presence of a PD-1^+^ NK cell subset in CB samples and the percentage of this subset was significantly higher than in AHD and mothers, while there are no substantial differences between mothers and AHD both in terms of expression and frequency of PD-1 (**Figures 1A, B**). Interestingly, the frequency of CB samples with a PD-1^+^ NK cell subset was more than double that of AHD’s and mothers’ samples (52% versus 25% or 23% respectively) (**Figure 1B**).

The HCMV seropositivity rates of the mothers at delivery was 60%, while that of AHD was 48% (**Figure 1B**). As expected, all the AHD and mothers with a PD-1^+^ NK subset were seropositive for HCMV, a variable fraction of HCMV^+^ individuals (both ADH and mothers) were PD-1^neg^ (23% in ADH versus 37% in mothers) and all the HCMV^-^ individuals were negative for PD-1.

Given that none of them had had HCMV infection or reactivation during pregnancy, we then wondered if there was a correlation between PD-1 expression on CB-NKs and the mother’s HCMV seropositivity. However, the answer proved negative as PD-1 was expressed on both the NKs of newborns with HCMV positive (30%) and HCMV negative mothers (22%) (**Figures 1B, C**).

As in AHD samples, PD-1 was expressed on CD56^dim^ and CD56^neg^ cells, but not on CD56^bright^ cells, although we did find some rare exceptions as shown in **Figures 1D, E**.

### 3.2 Characterization of PD-1^+^ cord blood NK cell subset

In analyzing the distribution of the main NK cell receptors, and whether they’re able to deliver either inhibitory (KIRs, NKG2A, LILRB-1, Siglec-7) or activating (NKG2C, NKp30, NKp46) signals on PD-1^+^ and PD-1^-^ CD56^dim^ and CD56^neg^ CB-NKs cell subsets, we observed significant differences among PD-1^+^ CB-NKs, PD-1^-^ CB-NKs and PD-1^+^ AHD-NKs (**Figure 2**).

We confirmed that the CB-NK cell receptor repertoire was dominated by the expression of markers of immature state of NKs (i.e. high NKG2A; low KIRs and LILRB-1 and virtually no expression of CD57) compared to AHD (**Figure 2** and not shown). However, compared to PD-1^-^ CB-NKs, the PD-1^+^ CB-NK cell subset was characterized by a more mature phenotype, as shown by a higher fraction of KIRs^+^ and minor fraction of NKG2A^+^ cells. In addition, Siglec-7 was significantly under expressed on PD-1^+^ CB-NKs compared to PD-1^-^ CB-NKs. Notably, although CB showed a reduced expression of the NKG2C activating receptor, PD-1^+^ CD56^dim^ NKs were characterized by an increased expression of NKG2C as compared to PD-1^-^ CD56^dim^ NKs. Regarding main activating receptors, PD-1^+^ NKs showed lower levels of both NKp30 and NKp46, under expression that was more evident on PD-1^+^ CD56^neg^ NKs.

Since we previously showed that in AHD the PD-1^+^ NK cell subset is characterized by a KIR^+^NKG2A^-^CD57^+^NKp46^low^ phenotype (1), we compared PD-1^+^ CB-NK cell subset with PD-1^+^ AHD-NK cell subset. Interestingly, PD-1^+^ CB-NKs co-expressed both NKG2A and KIRs while PD-1^+^ AHD-NKs did not co-express NKG2A. As mentioned before, CB-NKs express low levels of LILRB-1. Regarding Siglec-7, this inhibitory receptor was under expressed on PD-1^+^ CB-NKs as compared with PD-1^-^ CB-NKs, although this under expression was even more evident on PD-1^+^ AHD-NKs. As expected, PD-1^+^ CB-NKs showed very reduced levels of CD57 compared to PD-1^+^ AHD-NKs. The NKG2C receptor (the activating counterpart of NKG2A) was significantly decreased on CB-NKs compared to AHD-NKs primarily on PD-1^+^CD56^neg^ CB-NKs (**Figures 2A–D**).

Interestingly, although NCRs were under expressed on PD-1^+^ CB-NKs as compared to PD-1^-^ CB-NKs, both NKp46 and NKp30 were up-regulated on PD-1^+^ CB-NKs compared to AHD-NKs and this was particularly evident on the PD-1^+^CD56^dim^ subset (**Figures 2C, D**).

In summary, in CB, PD-1^+^ NKs while mostly exhibiting a mature signature, also tend to express NKG2A, in contrast with PD-1^+^ AHD-NKs. This hints to the fact that this receptor may appear early on maturing NKs (**Figures 2C, D**).

For this reason, we also analyzed CB-derived CD34^+^ stem cells (**Figure 2E**) as well as CD34^+^ cells from adults’ peripheral blood (not shown) for PD-1 expression. In all experiments performed, CD34^+^ cells were virtually negative for PD-1 expression, PD-1 being present in 0.8% of CD34^+^ cells at most.

### 3.3 Immune checkpoint coexpression on PD-1^+^ and PD-1^-^ CB-NKs

**Figure 4:**
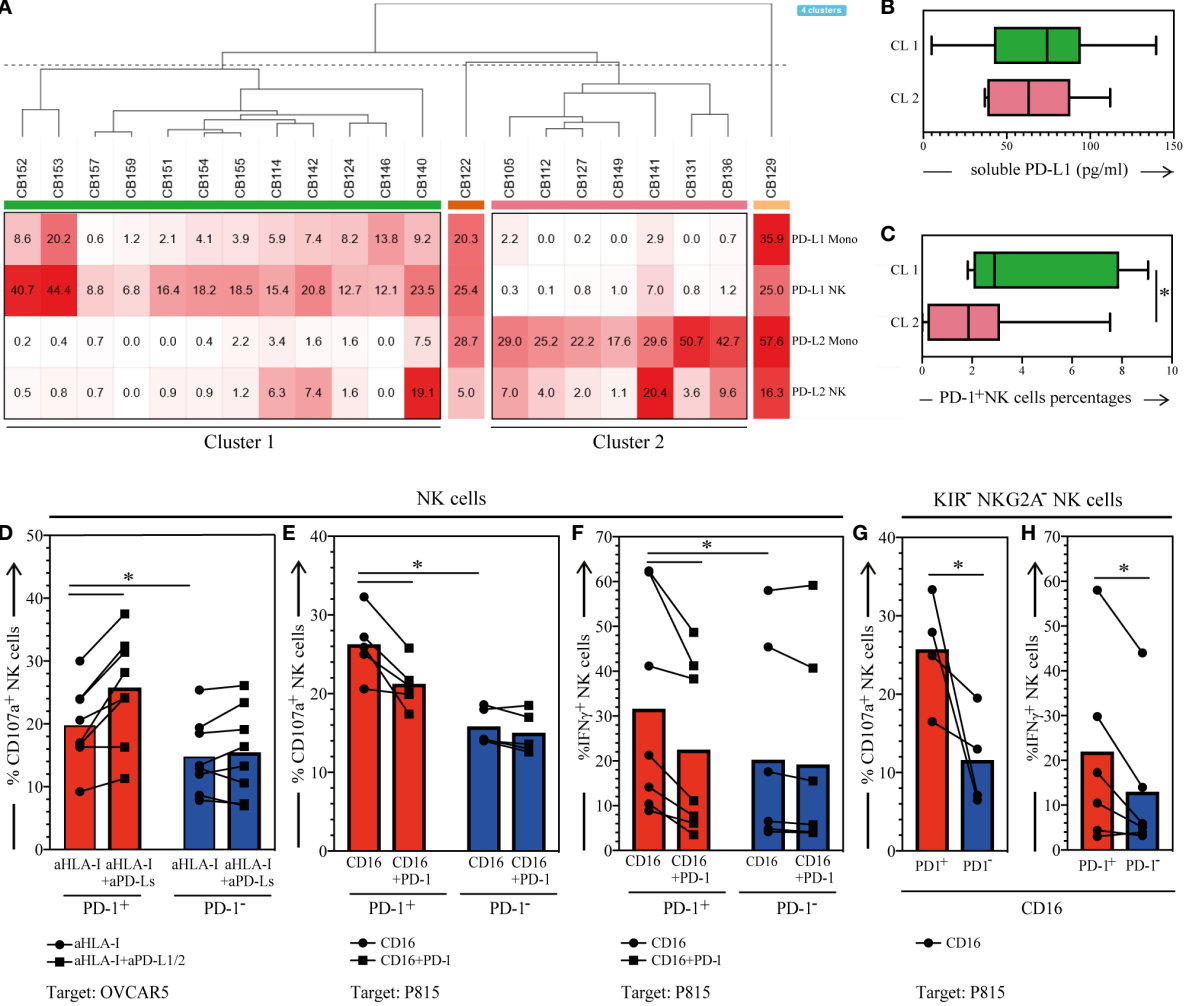
Membrane-bound/soluble PD-1 Ligands expression in the CB, and functional analysis of PD-1^+^ CB-NKs. **(A)** Hierarchical clustering of CB samples based on PD-L1 and PD-L2 expression on NKs and monocytes (N=21); **(B)** Soluble PD-L1 detected in the plasma of CB samples belonging to cluster 1 or cluster 2 **(C)** PD-1 expression on CB-NK cells belonging to cluster 1 or cluster 2; **(D)** CD107a expression on PD-1^+^ (red bars) and PD-1- (blue bars) CB-NKs after 3h coincubation with OVCAR5 in the presence of anti-HLA-I (aHLA-I) or anti-HLA-I plus anti-PDL-1 and anti-PD-L2 mAbs (aHLA-I+aPD-Ls) (n=8); **(E)** CD107a expression on PD-1^+^ (red bars) and PD-1- (blue bars) CB-NKs after 3h coincubation with FcγR^+^ P815 in the presence of anti-CD16 mAbs (CTR) or anti-CD16 and anti-PD-1 mAbs (PD-1) (N=5); **(F)** IFNγ production on PD-1^+^ (red bars) and PD-1- (blue bars) CB-NKs after 3h coincubation with FcγR^+^ P815 in the presence of anti-CD16 mAbs (CTR) or anti-CD16 and anti-PD-1 mAbs (PD-1) (N=7); **(G)** CD107a expression on KIRs^-^NKG2A^-^ PD-1^+^ (PD-1^+^, red bars) and PD-1^-^ (PD-1^-^, blue bars) CB-NKs after 3h coincubation with FcγR^+^ P815 in the presence of anti-CD16 mAbs (N=4); **(H)** IFNγ production on KIRs^-^NKG2A^-^ PD-1^+^ (PD-1^+^, red bars) and PD-1^-^ (PD-1^-^, blue bars) CB-NKs after 3h coincubation with FcγR^+^ P815 in the presence of anti-CD16 mAbs (N=6); Gating Strategy: **(A–C)** VI; **(D–F)** V; **(G, H)** VII. P value of less than 0.05 (*), was considered statistically significant; when not indicated, data were not statistically significant.

By exploring in more detail, the distribution of HLA-specific inhibitory receptors (KIRs, NKG2A, LILRB-1) on PD-1^+^ and PD-1^-^ CB-NKs, we generated a t-SNE algorithm from an equal number of PD-1^+^ and PD-1^-^ CB-NKs derived from 15 different CBs (**Figure 4**). This representation highlighted four distinct PD-1^high^ NK cell populations characterized by all combination of KIRs and NKG2A (**Figure 4A** first column and panel B).

Notably, as shown in **Figure 2**, in CB, PD-1^+^ NKs co-expressed not only KIRs, but also NKG2A. In particular, we detected: 1) a subpopulation expressing only KIRs (36% of PD-1^+^ NKs; **Figure 4**, purple gate); 2) a subpopulation co-expressing KIRs and NKG2A (16% of PD-1^+^ NKs; **Figure 4**, black gate); 3) a subpopulation expressing NKG2A, but not KIRs (21% of PD-1^+^ NKs; **Figure 4**, yellow gate); 4) a subpopulation not expressing either (18% of PD-1^+^ NKs; **Figure 4**, light blue). Additionally, we showed the presence of a discrete subset of CB-NKs that expressed PD-1 but was negative for all classical NK inhibitory receptors including KIRs, NKG2A and LILRB-1 (**Figure 4** last column).

### 3.4 PD-1 is functional and mediate negative signals in CB-NKs

To better understand if PD-1 expression on CB-NKs could be due to an education process, we explored the expression of PD-1 ligands (PD-L1/PD-L2) on CB immune cells and the functional features of PD-1^+^ CB-NKs.

We found that CB-T and B lymphocytes are characterized by low/negative expression of PD-L1 and PD-L2 on their surface (not shown), whereas NKs and monocytes can express both PD-L1 and PD-L2, hence suggesting possible cis and/or trans PD-1/PD-Ls interactions in healthy conditions. Particularly, upon analyzing CB-immune cells by unsupervised hierarchical clustering, we found two clusters clearly separating CBs characterized by NKs and monocytes expressing PD-L1 (cluster 1) from those expressing PD-L2 (cluster 2) (**Figure 3A**, **Figure S2**). Variable levels of soluble PD-L1 were detected in nearly all CB-derived plasma analyzed with no difference between the two clusters (**Figure 3B**). The PD-L soluble form was correlated neither with PD-1 expression on CB-NKs nor with PD-Ls expression on NKs and monocytes (not shown). Also regarding surface molecules, we found no correlation between PD-L1/PD-L2 and PD-1 expression on the cell surface of CB-immune cells (data not shown), however, we found a significantly higher expression of PD-1 on NKs belonging to cluster 1 compared to the NKs of cluster 2 (**Figure 3C**).

We then performed functional analysis of PD-1^+^ CB-NKs to evaluate if, in CB, PD-1, can deliver an inhibitory signal, upon interactions with PD-Ls^+^ target cells or when triggered with specific anti-PD-1 mAb.

First, we analyzed the effect of engagement of PD-1 by its ligands (PD-L1/PD-L2) expressed on the human ovarian carcinoma cell line OVCAR5. In these experiments the expression of CD107a on NK cells was analyzed after short-term exposure of CB-NKs to OVCAR5 in the presence or absence of anti–PD-L1/PD-L2 mAbs, which can block the interaction between PD-1 and its ligands (mAb-mediated masking experiments). As shown in **Figure 3D**, PD-1^-^ CB-NKs displayed a visible mobilization of CD107a, both in the absence and presence of anti–PD-L1/PD-L2 mAbs. Most importantly, degranulation of PD-1^+^ CB-NKs was higher than that of PD-1^-^ CB-NKs in the absence of anti–PD-L1/PD-L2 mAbs and could be further increased by anti–PD-L1/PD-L2 mAbs. Note that we performed experiments in the presence of anti–HLA class I mAbs (A6/136) to disrupt the inhibitory interaction between KIRs and HLA class I molecules (expressed at high levels on the surface of OVCAR5) (1).

Then, PD-1^+^ CB-NKs were assessed for degranulation capability in a reverse antibody-dependent cellular cytotoxicity (R-ADCC) against the murine FcγR^+^ P815 mastocytoma cell line (PD-L1/PD-L2 negative). These experiments were performed in the presence of anti-CD16 mAb and in the presence or absence of anti-PD-1 mAb. The aim was to assess whether mAb-mediated cross-linking of PD-1 could inhibit NK cell activation induced by the mAb-mediated cross-linking of CD16. We observed that mAb-mediated cross-linking of PD-1 resulted in a significant reduction of PD-1^+^ NK cell degranulation (**Figure 3E**). Nevertheless, once again PD-1^+^ CB-NKs displayed a significatively higher degranulation compared to PD-1^-^ CB-NKs in the absence of PD-1 triggering. This clearly indicates that the expression of PD-1 provides an additional level of inhibition of the functional activity of CB-NKs. In addition, to quantify the possible contribution of PD-1 to CB-NK cell education, we gated on all KIRs^-^NKG2A^-^ NKs and compared the percentage of CD107a^+^ cells upon NK cell stimulation between PD-1^+^ and PD-1^−^ subsets. CD107a expression was greater within PD-1^+^ CB-NKs than within PD-1^−^ CB-NKs (**Figure 3F**). Of note, the superior functional response of PD-1^+^ cells does not depend on differences in activating CB-NK receptor repertoire since PD-1^+^ CB-NKs express lower levels of NKp46 (whose ligand is expressed on P815 cells) and similar levels of CD16 compared to PD-1^-^ CB-NKs.

### 3.5 PD-1 expression increase IFN-γ production capacities

CB-NK cells were also evaluated for cytokine release. Similar to degranulation, IFN-γ production could be induced in PD-1^+^ NK cells by anti-CD16 mAbs in a redirected-antibody dependent stimulation assay (P815 cells plus mAb) (**Figure 3G**). Notably, this release was significantly higher than that by PD-1^-^ CB-NKs (**Figure 3G**). On the other hand, when anti-CD16 was combined with anti–PD-1 mAb, a significantly lower fraction of PD-1^+^ NK cells produced this cytokine. Also in this case, to determine the possible contribution of PD-1 to CB-NK cell education, we gated on all KIRs^-^NKG2A^-^ NKs and compared the percentage of IFN-γ^+^ cells upon NK cell stimulation between PD-1^+^ and PD-1^−^ subsets. IFN-γ production was greater within PD-1^+^ CB-NKs than within PD-1^−^ CB-NKs (**Figure 3H**). These data further support the possibility that PD-1 can contribute to the CB-NK cell education process.

## 4 Discussion

Here, we analyzed the phenotypic and functional features of NKs in newborns’ CB. Our results demonstrate that, at birth, we can find some newborns with functional PD-1 on NKs, co-expressing NKG2A and KIRs.

Interestingly, no previous studies have reported the expression of PD-1 on a subpopulation of fresh isolated CB-derived NKs in normal physiological conditions, apart from a single study that reported the expression of PD-1 on expanded stimulated CB-NKs and AHD-NKs, without any characterization of the PD-1^+^ NK cell subset (17).

Recent studies described CB-NKs to represent about 30% of total CB lymphocytes (18). These cells when expanded *in vitro* are capable of secreting higher levels of IFN-γ and expressing higher levels of CD69 upon stimulation when compared to the AHD-NKs. They and others showed that in newborns (particularly in CB), NK cell receptor repertoires are dominated by the expression of NKG2A. In addition, CB-NKs are characterized by a lower expression of KIRs, DNAM-1, NKG2C, IL-2R, and granzyme B, hence, possessing less cytotoxic abilities when compared to AHD-NKs. However, upon stimulation with cytokines, CB-NK cytotoxicity was comparable to AHD-NKs.

Studies reporting the changes in the gradual expression of HLA-specific inhibitory receptors showed that the expression and frequency of NKG2A decreases significantly during infancy with no further significant decrease beyond the second decade of life. In contrast, the expression of KIRs is very low at birth, and shows a significant increase during the first decade with a sharp rise as early as the first two years of life. Importantly, no further significant increase in KIRs expression is observed in adults (13). Therefore, it suggests that the main changes in receptor repertoires in NKs take place during infancy and puberty, at least as far as HLA-I-specific inhibitory receptors are concerned. However, further studies and general information on the health, vaccination, and serology of individuals are needed to further substantiate these observations. For example, it is known that, in a fraction of individuals, the KIR/NKG2A balance might be disrupted by HCMV infection, which, as we mentioned, leads to the generation of the so-called adaptive NKs (12, 13, 19).

In this study, we attempted to identify the impact of the potential role of PD-1/PD-L1 interactions in controlling NK cell development, education and activity. This stemmed from the fact that recently the existence of a subpopulation of NKs expressing high levels of this receptor has been demonstrated in AHD-NKs (18-60 years of age), confirming also that its expression impairs NK cell function against PD-L^+^ tumor targets. Specifically, we observed that this receptor was present in one quarter of the analyzed AHD population (1), although it has been recently showed that PD-1 mRNA and a cytoplasmic pool of PD-1 protein could be detected in resting, surface PD-1^-^, NK cells from AHD (20). In AHD, surface PD-1 expression was confined to CD56^dim^ NKs and, if present, on CD56^neg^ NKs, whereas the CD56^bright^ subset was consistently PD-1^-^. The percentage of PD-1 varied from donor to donor and, in rare cases, reached very high values (50%). Moreover, PD-1 was confined to terminally differentiated NKs characterized by the KIR^+^NKG2A^-^LILRB-1^+^CD57^+^ phenotype (which is the same population of more mature CD57^+^ NKs generally expanded in HCMV infections). In this context, further analysis allowed us to highlight the existence of a direct correlation between HCMV infection and the presence of PD-1 on NKs in the AHD analyzed. Indeed, all individuals with PD-1^+^ NKs were found to be seropositive for HCMV. Furthermore, as expected, PD-1 positive individuals exhibited a reconfiguration of the NK cell receptor repertoire, typically induced by HCMV infection; in particular, the NKs of these individuals were characterized by an up-regulation of the activating receptor NKG2C and an under expression of the inhibitory receptor Siglec-7. This means that, at least in AHD, PD-1 expression may be rapidly induced on NK cells following HCMV-mediated activation and this expression could be maintained in the setting of chronic virus infection and has been associated with a progressive loss of NK cell functions, similar to what happens to T lymphocytes.

To understand whether PD-1 could be expressed only by HCMV^+^ adults or also at earlier times and in a HCMV-independent manner, we analyzed NKs in newborns (specifically in CB).

By analyzing the expression of PD-1 on CB-NKs, we found that this receptor was expressed on more than half of the samples analyzed. Since PD-1 receptor may be expressed on NKs also in immature context as CB, we also analyzed CB-derived CD34^+^ stem cells for PD-1 expression. In all experiments performed, CD34^+^ cells were virtually negative for PD-1 expression, this receptor being consistently expressed in less than 1% of CD34^+^ cells.

Regarding CB-NKs, PD-1 was expressed on CD56^dim^ and CD56^neg^ NKs, which are present in significant amounts in CB, and almost never on CD56^bright^ cells, although we found a few rare exceptions as shown in **Figure 1**.

Thanks to further detailed analysis of different markers on PD-1^+^ and PD-1^-^ CB-NKs, we observed that, in general, PD-1^+^ CB-NKs, compared with PD-1^+^ AHD-NKs, in addition to KIRs, can co-express NKG2A, and very little LILRB-1 and CD57 (this is mainly due to the more immature signature of CB-NKs). Finally, PD-1^+^ CB-NKs express higher levels of the activating receptors NKp30 and NKp46 compared to PD-1^+^ AHD-NKs (**Figure 2**).

We did not find any correlation between PD-1 expression on maternal NKs and PD-1 expression on CB-NKs. Interestingly we observed a significant increase in frequency of PD-1 expression on NKs going from PB of HCMV^+^ AHD and mothers to CB (**Figure 1**).

We then analyzed whether there was a correlation between maternal HCMV seropositivity and PD-1 expression on CB-NKs, especially given the results on AHD discussed earlier, which indicated that PD-1 was present only on NKs of HCMV^+^ individuals. Of note, CB samples received were from mothers whose HCMV seropositivity rates at delivery were approximately of 60% and who had not had HCMV infection or reactivation during pregnancy. Our data indicates the absence of a correlation between maternal seropositivity for HCMV and the presence of PD-1 on newborns’ NKs, as shown in **Figure 1**. In fact, PD-1 was also expressed on NKs from newborns with HCMV-seronegative mothers.

Thus, we assume that PD-1 could appear early during NK maturation on the more immature NKG2A^+^ cells then it moves on to the more mature KIR^+^ cells. At birth, we can find some newborns with PD-1 on NK cells, co-expressing NKG2A and KIRs. Note that this expression is completely independent of HCMV. However, we still don’t know if this expression is stable over time or whether this depends on additional factors/stimuli. What we do know though is that in AHD, PD-1 is expressed only on a part of the population that was found to be HCMV^+^ and only on terminally differentiated NK cells (KIR^+^, NKG2A^-^).

Recently, PD-1 expression was detected early in the development of some ILC subsets and in the fetal-maternal compartments, which was thought to play a role in the acquisition of ILC effectiveness and tolerance (21, 22). Furthermore, lack of PD-1 has been shown to affect T and B cell development, as well as maturation (23, 24). These data raise the question of whether and how PD-1 is involved in NK cell development and education, and how the presence of PD-1 may affect NK cell functions.

Here we show that PD-1 is functional since its engagement can send negative/inhibitory signals upon interaction with PD-L1 and PD-L2 expressing target cells. In addition, PD-1 can inhibit the activation of NKs mediated by CD16-triggering both in terms of degranulation and IFN-γ production. Yet, in CB-NKs lacking both KIRs and NKG2A, PD-1^+^ NKs showed higher response to stimulus when PD-1 was not triggered, with increased natural cytotoxicity and enhanced IFN-γ production as compared to PD-1^-^, KIRs^-^, NKG2A^-^ CB-NKs. This supports the claim that early PD-1 expression on CB-NKs can contribute to the NK cell education process.

The fact that PD-1^+^ CB-NKs express lower levels of NCRs (whose ligands are expressed on PD-Ls^+^ target cells used in our experiments) further support the hypothesis that the higher functional response of PD-1^+^ cells more likely arises from PD-1-mediated education or from differences in maturation.

It remains to be clarified whether the expression of PD-1 on NK cells is not related to peculiar arrangements of NK receptor repertoire in everyone, for example the presence of few educated KIRs or activating KIRs (25). In this context, it could be interesting to explore whether PD-1 expression is more frequent in individuals with a specific haplotype. It will be important to further investigate this aspect.

Differently, in AHD, PD-1^+^ NK cells can expand in the context of chronic immune activation and exclusively on terminally differentiated (NKG2A^neg^, CD16^high^) NK cells and this suggests that PD-1 might contribute to modulate NK cell functional activity by decreasing natural cytotoxicity but enhancing ADCC, during infections such as HCMV (26).

There is accumulating evidence that NKs participate in the therapeutic effects of mAbs against PD-1 or PD-L1, especially against tumors with low HLA-I expression. PD-1 and PD-L1 have recently been shown to form cis-interactions in artificial lipid structures and in antigen-presenting cells (APCs) (27). In this context, our data showing that PD-L1 molecules are expressed in the membrane of NKs and monocytes, suggest that PD-1 and PD-L1 may form cis interactions with each other on NKs or trans interactions with monocytes, thus affecting the NK cell response to PD-1 signaling. A recent study has shown that NKs up-regulate PD-L1 in response to IFN-γ (28). PD-L1^+^ cells negatively regulate PD-1^+^ effector cells, but at the same time, PD-L1 on NKs might inhibit survival of PD-1^+^ APCs (29). In the case of CB, in addition to binding PD-1 in cis, PD-1 can also bind to PD-Ls expressed on the monocytes’ membrane. These multi-facetted binding patterns in cis and trans may contribute to the fine tuning of the immune response within the compartments in which NKs develop and become educated.

Recent findings (30) suggest that the inherited PD-1 deficiency patient’s lymphocytes produce only small amounts of interferon IFN-γ upon mycobacterial stimuli (31) and that without appropriate PD-1 signaling, activated lymphocytes may become vulnerable to exhaustion and deletion. Overall, the concurrent autoimmunity and severe immunopathological phenotypes in multiple vertebrates with inherited or acquired PD-1 deficiency suggests that PD-1/PD-Ls interactions are evolutionarily indispensable for both self-tolerance and protective immunity (32).

In summary, in CB, PD-1 can be expressed on NKs independent of HMCV infection, it can also be expressed on more immature NKG2A^+^CD57^-^ cells, hinting that this receptor may appear early on maturing NKs, and it is functional. Thus, it is yet to be explored in depth whether PD-1 can contribute to the NK cell education program in the early stages of NK cell differentiation, at least in some individuals, and/or if its early expression can impact NK cell effector functions.

PD-1 blockade immunotherapy is now widely used in the treatment of different cancer conditions (4–6). Our findings suggest that PD-1 blockade can also be used to modulate NK cell differentiation/education process or be beneficial in enhancing the effector functions of NKs.

Primary NK cell sources used in clinical trials to treat both hematologic malignancies and solid tumors include cells derived from CB (33). Considering the value of these cells in clinical practice, the study of their properties may prove useful to obtain a better use of them and to improve their clinical efficacy.

In the future, it will be important to fully understand if the expression of PD-1 and other co-expressed checkpoint inhibitors may affect the clinical use of these cells and impact the selection of the most suitable CB-derived NK cell populations for therapeutic applications.

## Data availability statement

The original contributions presented in the study are included in the article/**Supplementary Material**. Further inquiries can be directed to the corresponding authors.

## Ethics statement

The studies involving human participants were reviewed and approved by the ethics committee of the Liguria Region, Genova, Italy (Prot. n. 39/2012, number CER Liguria: DB id 10125). This study was carried out in accordance with the recommendations of the ethical standards of the institutional and/or national research committee. The patients/participants provided their written informed consent to participate in this study.

## Author contributions

MG designed, performed research, interpreted data, and wrote the article VA, AB, and GU selected and provided samples and VO performed research and interpreted data. FR, RG, interpreted data and revised the article. SC, MC, SS, revised the article. SP designed, performed research, interpreted data, and wrote the article. EM designed and performed research, interpreted data, wrote and financed the article. All authors contributed to the article and approved the submitted version.
