# Supplementary material for: Identification of a novel cord blood NK cell subpopulation expressing functional programmed death receptor-1
Source: Front Immunol. 2023 Jun 22;14:1183215. doi: 10.3389/fimmu.2023.1183215 (PMC10335745; doi:10.3389/fimmu.2023.1183215)
Supplement: Supplementary file 1 [file DataSheet_1.pdf]

Supplementary Material

1.1 Supplementary Figures

Figure S1.

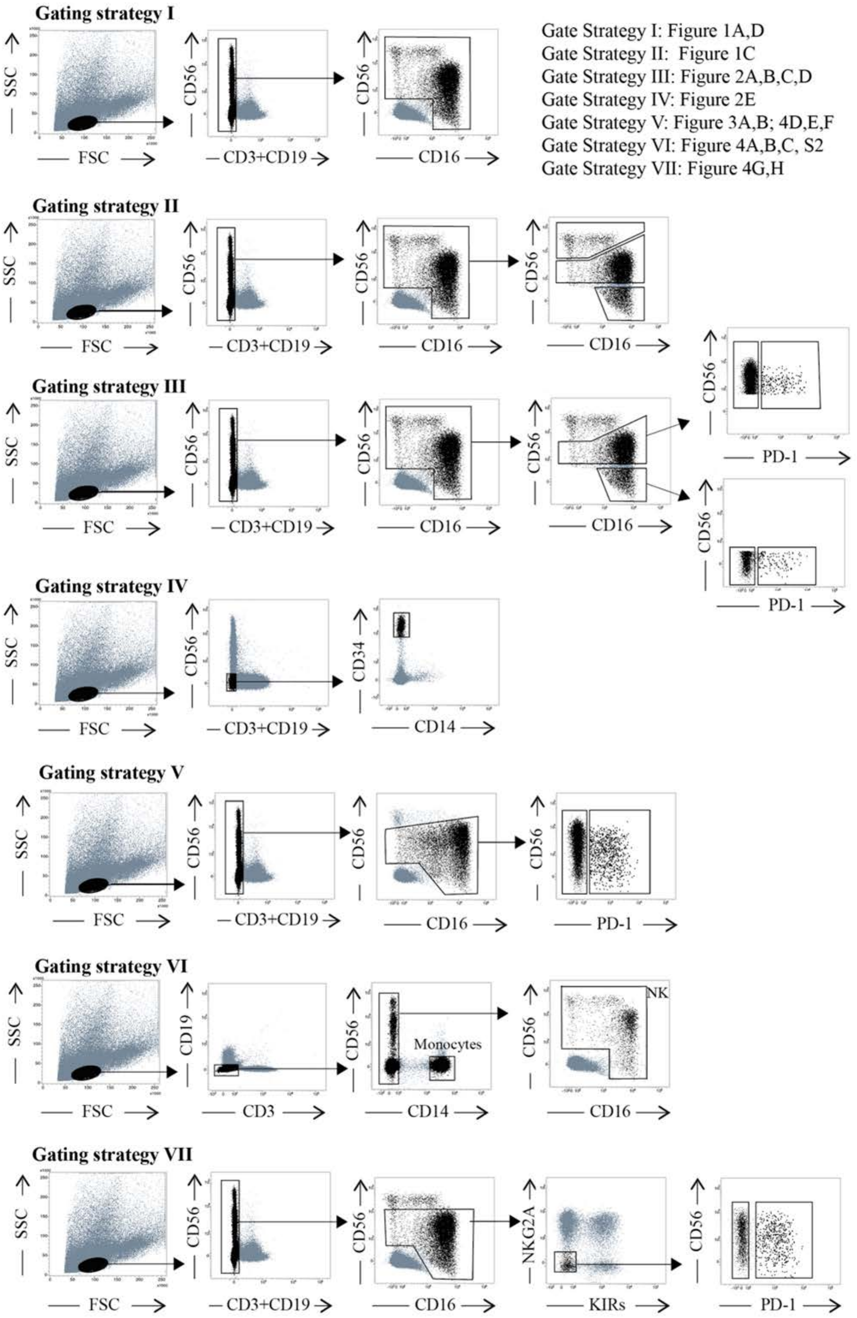

Gating Strategy. Illustration of all gating strategies adopted for the figures, relevant panel for each gating strategy are indicated in the top right

Figure S2.

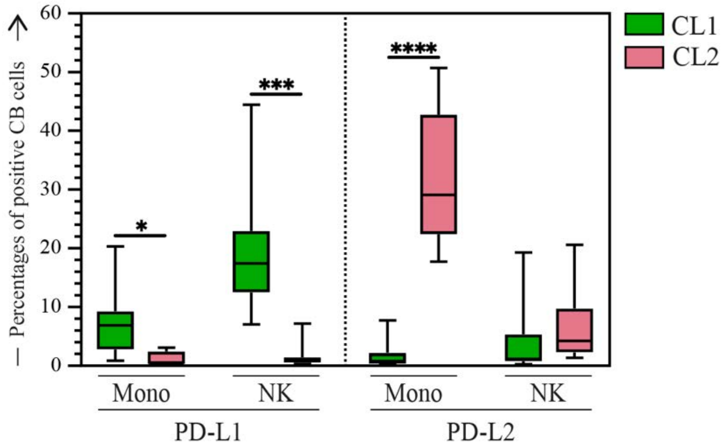

PD-L1 and PD-L2 expression in the two clusters. Box and whiskers graph illustrating the different expression of PD-L1 and PD-L2 in Monocytes and NK cells between the two clusters of CB identified in Figure 4A
